# A human-LLM collaborative annotation approach for screening articles on precision oncology randomized controlled trials

**DOI:** 10.1186/s12874-025-02674-3

**Published:** 2025-09-29

**Authors:** Hui Chen, Jiale Zhao, Sheng Zheng, Xinyu Zhang, Huilong Duan, Xudong Lu

**Affiliations:** College of Biomedical Engineering and Instrument Science, Zhejiang University, Hangzhou, 310058 China

**Keywords:** Human-LLM collaboration, Article screening, Precision oncology randomized controlled trials

## Abstract

**Background:**

Supervised learning can accelerate article screening in systematic reviews, but still requires labor-intensive manual annotation. While large language models (LLMs) like GPT-3.5 offer a rapid and convenient alternative, their reliability is challenging. This study aims to design an efficient and reliable annotation method for article screening.

**Methods:**

Given that relevant articles are typically a small subset of those retrieved articles during screening, we propose a human-LLM collaborative annotation method that focuses on verifying positive annotations made by the LLM. Initially, we optimized the prompt using a manually annotated standard dataset, refining it iteratively to achieve near-perfect recall for the LLM. Subsequently, the LLM, guided by the optimized prompt, annotated the articles, followed by human verification of the LLM-identified positive samples. This method was applied to screen articles on precision oncology randomized controlled trials, evaluating both its efficiency and reliability.

**Results:**

For prompt optimization, a standard dataset of 200 manually annotated articles was equally divided into a tuning set and a validation set (1:1 ratio). Through iterative prompt optimization, the LLM achieved near-perfect recall in the tuning and validation sets, with 100% and 85.71%, respectively. Using the optimized prompt, we conducted collaborative annotation. To evaluate its performance, we manually reviewed a random sample of 300 articles that had been annotated using the collaborative annotation method. The results showed that the collaborative annotation achieved an F1 score of 0.9583, reducing the annotation workload by approximately 80% compared to manual annotation alone. Additionally, we trained a BioBERT-based supervised model on the collaborative annotation data, which outperformed the model trained on data annotated solely by the LLM, further validating the reliability of the collaborative annotation method.

**Conclusions:**

The human-LLM collaborative annotation method demonstrates potential for enhancing the efficiency and reliability of article screening, offering valuable support for systematic reviews and meta-analyses.

## Background

In the research process of systematic reviews and meta-analyses, article screening plays a crucial role. This process involves the detailed evaluation of a large volume of articles to identify documents highly relevant to a specific research topic. The purpose of article screening is to ensure that the articles included in the study are of high quality, effectiveness, and relevance, thereby laying a solid foundation for the research [1]. Manual screening of articles is a time-consuming and complex task, especially when dealing with thousands of documents. Although only a small proportion of the articles meet the research criteria [2], researchers still need to review each document individually. To alleviate this burden, studies have utilized natural language processing (NLP) technologies, including traditional machine learning models (such as CNN [3], LSTM [4]) and pre-trained language models (such as BERT [5–7]), for automatic article classification. However, these models, mostly based on supervised learning, still require a significant amount of manually annotated data for training.

Recently, the development of large language models (LLMs) has provided new possibilities for automatic article screening. Many models can perform text annotation based on natural language instructions, greatly reducing the dependence on large-scale manually annotated datasets, and enabling researchers with limited programming knowledge to conduct large-scale automatic text analysis [8–10]. For instance, Hu et al. [11] investigated the use of ChatGPT for zero-shot information extraction from radiological reports, demonstrating its potential to extract structured information without parameter tuning. Despite the significant advantages of LLMs in terms of convenience, accuracy, and cost, they still have some limitations. The potential for biased labels due to biased training data [12], as well as the difficulty in understanding the nuances of natural language in different contexts [13], leads to considerable variation in performance across different tasks, datasets, and labels. Therefore, human supervision and intervention remain essential in article screening.

Previous research [14] has indicated that human intervention is unavoidable when using LLMs for text annotation. The automated annotation process of LLMs requires verification of performance through human-generated labels and iterative optimization of prompts based on the model’s misclassifications. Furthermore, since LLM outputs are not always accurate, selective human verification of model outputs has become a crucial step in the text annotation process. The MEGAnno+ [15] annotation system developed by Hannah and colleagues evaluated classification confidence using the logit scores of the LLM’s responses and manually verifies annotations with a confidence level below 95%. Similarly, Hamidreza and Masoud [16] verified annotations with low confidence by querying the model about its confidence level in the prompt and comparing the performance of models trained with different confidence levels. However, the reliability of the confidence scores generated by the LLMs themselves has not been fully validated, and determining an appropriate confidence threshold remains a challenge.

**Fig. 1:**
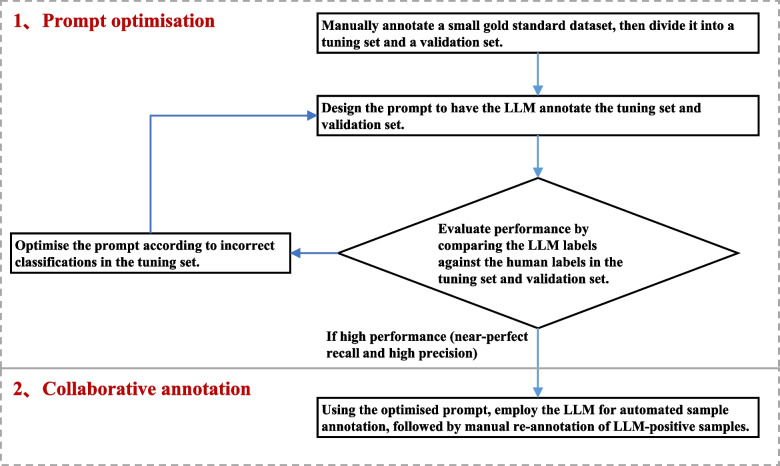
The workflow of human-LLM collaborative annotation

In this work, we consider that during the article screening process, the articles meeting specific criteria typically constitute only a small portion of the retrieved articles, i.e., the positive samples are few. Based on this observation, we hypothesize that if we could develop a model with near-perfect recall and high precision, then the negative samples annotated by the LLM would mostly be correct. Although there may be errors in the positive samples annotated by the LLM, their number is small, and we can effectively correct these errors through selective manual verification of these positive samples without imposing a heavy burden on human review. As illustrated in Fig. 1, our workflow for human-LLM collaboration is divided into two steps: First, we optimized the prompt using a manually annotated standard dataset, refining it iteratively to achieve near-perfect recall for the LLM. Second, the LLM, guided by the optimized prompt, annotated the articles, followed by human verification of the LLM-identified positive samples. This study used articles on precision oncology randomized controlled trials as a case study to evaluate the efficiency and reliability of the proposed method. The experimental results suggest that by optimizing the LLM’s prompt and manually verifying the positive samples, we can improve the reliability of the LLM’s annotations. This approach shows promise in supporting systematic reviews and meta-analyses.

## Methods

### Data source and screening criteria

In this study, we validated our method by screening articles on precision oncology randomized controlled trials (RCTs). Since there are no specific subject terms for “precision” in this context, we retrieved articles from PubMed [17] using the search query: “randomized controlled trial”[pt] AND “cancer”[MeSH Major Topic] AND “humans”[mh]. This search yielded 23,521 articles published between January 1, 2012, and December 31, 2023.

To identify articles on precision oncology RCTs from this set, we established four criteria as shown in Table 1: (1) the article must be a randomized controlled trial, (2) the study population must be cancer patients, (3) the study purpose must be cancer treatment evaluation, and (4)the study must involve biomarkers related to genetic and molecular characteristics. An article is considered a precision oncology RCT only if it meets all four criteria.

**Table 1:** The screening criteria for precision oncology RCTs

Name	Content
Format	Randomized controlled trial
Population	Cancer patients
Purpose	Cancer treatment evaluation
Biomarker	Genetic and molecular characteristics

During the manual annotation process, experts assessed whether each article met these criteria based on the title and abstract. To ensure accuracy and reliability, two experts conducted the initial annotations independently. Any discrepancies were reviewed by an additional annotator who made the final decision. We used the Medtator [18] annotation tool for this process.

### Design of ChatGPT prompt

Using the OpenAI API (https://platform.openai.com/docs/api-reference), we selected “gpt-3.5-turbo” as our base model. We utilized the role attribute in the message objects to define the prompts for “system” and “user” roles. The “system” role was employed to set the context and guidelines, providing ChatGPT’s virtual character and relevant background information. The “user” role described specific requirements, detailing the tasks and expected response format. In our API implementation, we configured the top-p parameter to its default value of 1, while setting the temperature to 0. This configuration minimizes the variability of the returned responses. For additional details on the API usage and settings, please refer to our code repository on GitHub.

**Fig. 2:**
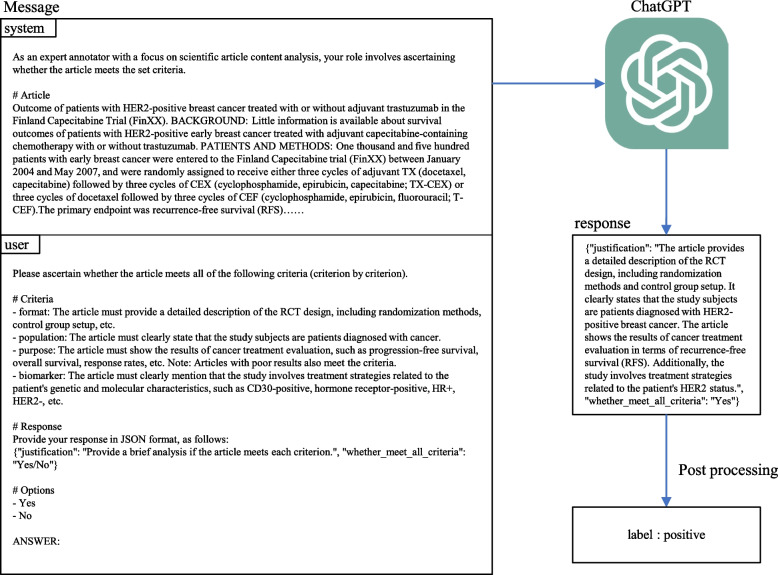
The schema for article screening using ChatGPT

For the task of screening articles on precision oncology RCTs, the prompt design in the “system” and “user” messages is illustrated in the Fig. 2. In the “system” message, the large model’s role was defined as “an expert annotator specializing in scientific article content analysis”, including specific content from scientific articles: the title and abstract. The “user” message specified the task of screening and annotation, requiring the determination of whether an article meets all criteria. We provided the expected response format in JSON, along with an example. To ensure consistent response formatting, we instructed the model to use yes/no options and appended “Answer:” after the article content to clarify the starting point of the response. This structure helps the model better understand where to generate the response, avoiding confusion when handling long inputs.

### Prompt optimization

To achieve near-perfect recall and high precision, we iteratively refined the LLM’s prompt manually using a standard dataset. The dataset was divided into a tuning set and a validation set, and the LLM was initially prompted to annotate both sets. Performance was assessed by comparing the labels generated by the LLM with those annotated by humans for both sets. If the performance metrics (recall and precision) were satisfactory, the process was concluded. If not, the prompt was revised based on an analysis of misclassifications in the tuning set. This cycle of evaluation and prompt refinement was repeated until the model demonstrated consistently high performance, characterized by near-perfect recall and high precision.

During the iterative prompt optimization process, we focused on three levels of refinement and adjustment for the LLM’s prompt. The first level addressed the structure of the prompt framework, such as whether the specific content of the article (title and abstract) should be included in the “system” message or the “user” message, and whether GPT should be required to provide reasoning for its answers. The second level addressed how to determine whether an article meets multiple criteria—whether to assess each criterion independently or simultaneously. The third level focused on refining the conceptual description of each criterion and providing corresponding examples when the concepts were ambiguous, enabling the model to accurately classify the articles.

### Collaborative annotation

It is important to emphasize that our collaborative annotation approach is specifically designed for tasks involving article screening with a low prevalence of positive samples, where articles meeting specific criteria comprise only a small fraction of the retrieved set. The collaborative annotation process is as follows: using the optimized prompt developed in Prompt optimization section, we employed the LLM to annotate the articles. Given the near-perfect recall achieved by our model, the negative samples identified by the LLM are almost entirely accurate. Although errors may occur among the LLM-annotated positive samples, they are relatively rare. By selectively verifying these positive samples manually, we can effectively correct any misclassifications. This combined approach of LLM pre-annotation followed by manual validation significantly reduces the overall workload for article screening.

### Fine-tuning of the supervised model

To further validate the reliability of the human-LLM collaboration annotation data, we trained a supervised model using the collaboratively annotated articles to assess its performance. We selected the BioBERT [19] model, known for its excellent performance in previous studies [6, 20], for fine-tuning to perform the classification task. During preprocessing, we concatenated the article titles and abstracts and then input them into the BERT model. This model generates probabilities for each category, and if the probability of a specific category exceeds a threshold, the article is assigned the corresponding label. We conducted hyperparameter tuning to enhance the model’s reliability and significance. By carefully selecting and adjusting hyperparameters such as learning rate, batch size, and regularization strength, we aimed to achieve accurate and meaningful results.

## Results

### Result of prompt optimization

**Fig. 3:**
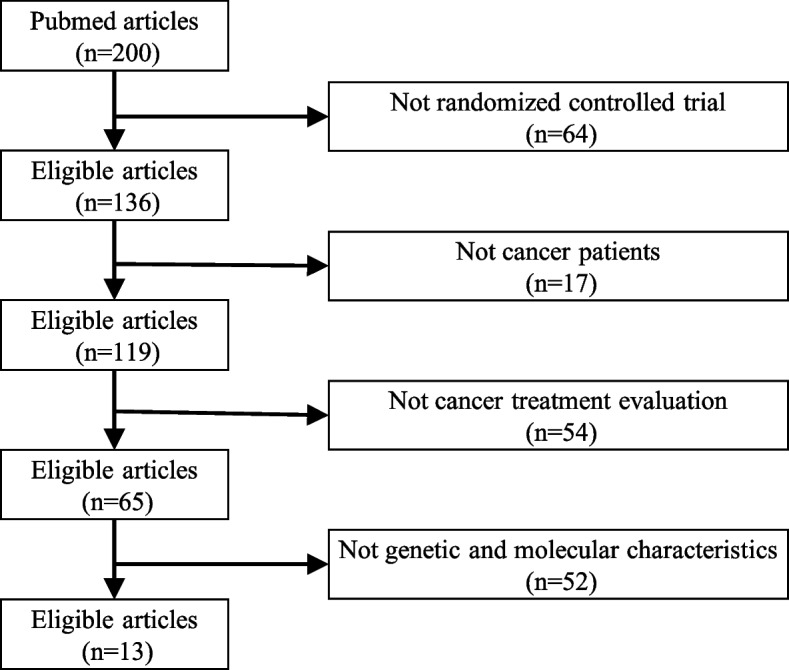
The descriptive statistics of the annotated corpus

To optimize the prompt and achieve near-perfect recall with the LLM, we first manually annotated 200 articles following the criteria and process described in Data source and screening criteria section. Each article was initially assessed by two annotators to determine whether it met each criterion. The inter-annotator agreement was evaluated using Krippendorff’s Alpha, which yielded a value of 0.65, indicating moderate agreement. The percentage of agreement was 96.50%, indicating a high level of overall consistency between the annotators. Any discrepancies were reviewed by an additional annotator who made the final decision. As shown in Fig. 3, we ultimately identified 13 precision oncology clinical trial articles.

We divided the 200 annotated articles equally into a tuning set and a validation set, each containing 100 articles. Based on the misclassifications in the tuning set, we optimized the prompts for the LLM. The final prompts, which performed well on both the tuning and validation sets, are detailed in Table 2. The optimization of LLM prompts is an iterative process, and the model’s performance does not always improve with each modification. It varies with different combinations of prompt optimization methods, making it challenging to present the iterative optimization process intuitively. However, based on our experience, the most significant impact on performance stems from the conceptual clarity of each criterion, followed by the method for determining whether an article meets multiple criteria, and finally, the structure of the prompt framework.

**Table 2:** The performance of the tuning and validation sets

	Precision	Recall	F1
Tuning set	0.3750	1.0000	0.5455
Validation set	0.4615	0.8571	0.6000

### Performance of human-LLM collaborative annotation

**Fig. 4:**
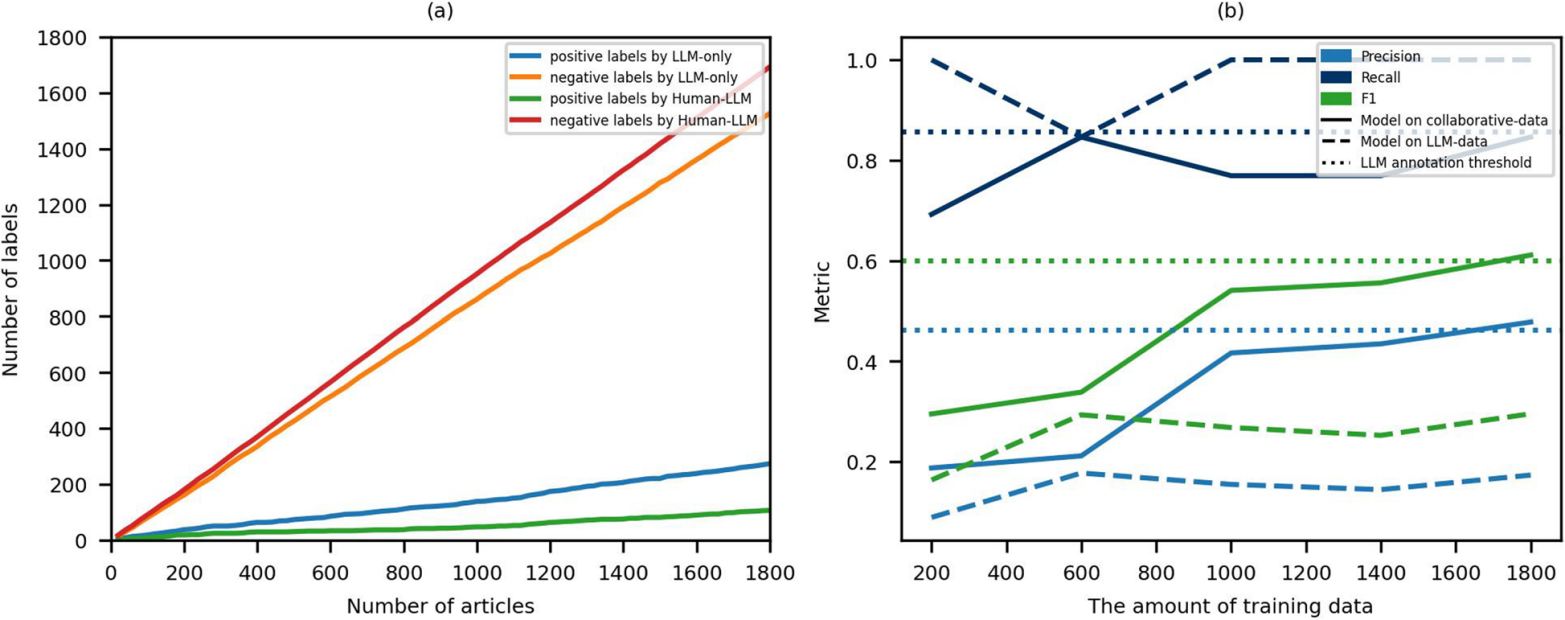
Comparison of collaborative annotation and LLM-only annotation. **a** Label Distribution Over Articles During Annotation Process, **b** The performance of the supervised model

Using the optimized prompt, we automatically annotated articles with the LLM and then articles identified as positive by the LLM underwent a secondary manual re-annotation. Figure 4a illustrates this annotation workflow. The blue line represents the number of positive labels initially assigned by the LLM, indicating the articles requiring further human review. As can be seen in Fig. 4, there are only 274 positive labels by the LLM for 1800 articles, which suggests that the pre-annotation of the LLM significantly reduces the number of articles that need to be manually annotated, decreasing the need for manual annotation by about 80% (1-274/1800). The green line shows the number of positive labels after human verification, illustrating the decrease in positive labels after correction. This decrease is attributed to the rectification of positive labels erroneously identified by the LLM, which were subsequently corrected to negative labels during the human verification phase.

To assess the accuracy of Human-LLM collaborative annotation,we randomly selected 300 articles from the collaboratively annotated dataset for manual review on an article-by-article basis to produce gold standard labels. The results of the comparison with the gold standard labels are shown in Table 3, where the F1 score of Human-LLM collaborative annotation is 0.9583, which is higher than the F1 score of annotating with the LLM only (0.6053). Table 4 presents the distribution of correct and incorrect classifications using the confusion matrix for both LLM-only annotation and human-LLM annotation. Compared to LLM-only annotation, human-LLM annotation improves annotation performance primarily by reducing the number of false positives through manual review (28 vs 0). These results indicate that human-LLM collaborative annotation improves the results of LLM-based annotation and that it has the potential to approach the performance of manual annotation.

**Table 3:** The performance of annotation on 300 articles

	Precision	Recall	F1
LLM-only annotation	0.4510	0.9200	0.6053
Human-LLM annotation	1.0000	0.9200	0.9583

**Table 4:** Confusion matrix for annotation performance on 300 articles

	LLM-only annotation		Human-LLM annotation	
	Positive	Negative	Positive	Negative
Human annotation Positive	23	2	23	2
Human annotation Negative	28	247	0	275

### Supervised model performance across different annotation strategies

We fine-tuned BioBERT based on LLM-annotated data and collaboratively annotated data, respectively. Each annotated dataset was split into training and test sets with a 4:1 ratio. The training set underwent negative sampling with a 5:1 ratio, meaning it contained five times more negative samples than positive ones. The test set consisted of 200 manually labeled articles. For training, we used a batch size of 8 and a learning rate of 5e-5. To prioritize higher recall, we selected models for testing that achieved a recall above 0.8 while maximizing the F1 score on the validation set.

Figure 4b illustrates the performance of the supervised model as the amount of training data increases. The model trained on collaboratively annotated data consistently outperforms the model trained on LLM-annotated data in terms of both precision and F1 score, despite showing slightly lower recall. This discrepancy may be attributed to the higher proportion of positive samples in the LLM-annotated data. Notably, the F1 score of the model trained on collaboratively annotated data is initially lower than that of the LLM annotation threshold when training data is limited. However, as the training data increases to 1,500 samples, the F1 score of the collaboratively annotated model surpasses that of the LLM annotation threshold. This result highlights the advantage of using a supervised model with a larger annotated dataset.

## Discussion

### Selection of article screening methods

Manual article screening ensures high-quality results but is time-consuming and labor-intensive, requiring skilled expertise. In contrast, the direct application of LLM for article screening offers advantages in speed and cost-effectiveness. However, the LLM may lack the nuanced understanding and contextual awareness provided by human annotators, which can lead to inaccuracies. A hybrid approach, combining the LLM with human annotation, can strike a balance between efficiency and accuracy. Specifically, the LLM can perform the initial screening, with human correction to enhance both efficiency and accuracy. Although this approach is more cost than relying solely on the LLM, it generally performs better in complex tasks that require deeper contextual understanding.

For long-term, large-scale article screening, or for tasks that need to be performed on an ongoing basis, training supervised models is a viable option. While developing supervised models requires a substantial amount of labeled data, their screening performance typically surpasses that of large models, reducing the need for manual corrections and thus proving more cost-effective in the long run. The annotated data necessary for supervised models can also be obtained through human-LLM collaborative annotation.

### Limitations and future work

The proposed human-LLM collaborative annotation method relies on the assumption that relevant articles constitute a small proportion of the search results. If this assumption is not met, the effectiveness of the method may be compromised. In cases where the proportion of relevant articles is higher, optimizing the LLM prompt for better precision, as well as manually verifying negative samples, may be necessary.

The difference in recall between the tuning set (100%) and the validation set (86%) during prompt optimization indicates potential overfitting. This is expected to some extent, as our method explicitly optimizes prompt to maximize recall on the tuning set. This design choice ensures that relevant instances are not missed, with human validation used to manage precision in subsequent stages. However, reducing overfitting remains an important area for future improvement. In future work, we plan to adopt two preliminary strategies to mitigate overfitting: (1) Designing prompts with broader and more inclusive criteria to better cover potential positives, even at the cost of lower precision and increased manual review burden; and (2) Increasing the size of the tuning set to improve the diversity and representativeness of samples, thereby enhancing prompt robustness and generalizability.

A key point of this approach is optimizing the prompt to achieve a high recall rate from the LLM. Our method focuses on manually verifying the positive annotations made by LLM to reduce false-negative samples and improve precision. We do not manually verify the negative annotations made by the LLM, and therefore cannot correct for false-positive samples. Therefore, the LLM must demonstrate high recall and strong generalization capabilities. Without these characteristics, the collaborative annotation method may result in a low recall rate, potentially omitting relevant literature. In the context of precision oncology article screening, such omissions could compromise the comprehensiveness of the retrieved evidence and affect the integrity of clinical decision-making.

Simultaneously, manually optimizing the prompt is a labor-intensive task. Although we provide guidance on optimizing prompts, focusing on refining the criteria for article screening, the inherent uncertainty in this process may lead to inefficiency and directional bias. Future work should focus on developing automated methods for prompt optimization to reduce the need for extensive manual intervention.

In this study, we used the API of ChatGPT for article screening. While the API is user-friendly and accessible, the underlying model may be subject to deprecation or changes over time. ChatGPT models have been shown to evolve, sometimes yielding different results for the same prompt due to back-end changes [21]. Therefore, to ensure reproducibility of the study, the recommended best practice is to employ an open source LLM based on publicly available training data.

Another challenge is the inherent class imbalance in the dataset, where negative samples far outnumber positive ones. Although the models achieve high overall accuracy, the precision of both the LLM and supervised models remains modest, highlighting the difficulty in identifying relevant articles within a predominance of irrelevant ones. Future research should explore strategies to mitigate this imbalance and enhance model performance in such scenarios.

Finally, while the proposed method was validated for screening precision oncology randomized controlled trial articles, its generalizability across various research areas remains to be tested. Additionally, we acknowledge the limitation of using a relatively small gold standard dataset. Although this dataset was sufficient for initial validation, a larger dataset would provide a stronger foundation for model tuning and yield more statistically robust conclusions. Future studies should extend this validation to different fields and employ larger datasets to confirm the method’s efficacy, adaptability, and reliability in diverse contexts.

## Conclusions

This study introduces an efficient and reliable Human-LLM collaborative method for article screening in systematic reviews and meta-analyses. By combining LLM annotations with manual validation, our approach achieves high accuracy while significantly reducing manual effort. When applied to screening of precision oncology randomized controlled trial article, the proposed method decreased the number of articles requiring manual annotation by approximately 80% compared to manual annotation alone, achieving an F1 score of 0.9583. In addition, a supervised model trained on collaboratively annotated data also performed well, outperforming a model trained on data only annotated by the LLM, further validating the effectiveness of the method. This method offers a promising solution for high-quality article screening that can reduce manual workload and support systematic reviews and meta-analyses across various research domains.

## Data Availability

Our dataset is available on GitHub, via https://github.com/Hui-zju/porct_as/tree/main/data.

## Abbreviations

LLM: Large language models
NLP: natural language processing
CNN: Convolutional neural network
LSTM: Long Short-Term Memory
BERT: Bidirectional Encoder Representations from Transformers
RCT: Randomized controlled trial
